# Development and validation of a prognostic model based on immune variables to early predict severe cases of SARS-CoV-2 Omicron variant infection

**DOI:** 10.3389/fimmu.2023.1157892

**Published:** 2023-03-01

**Authors:** Tianyu Lu, Qiuhong Man, Xueying Yu, Shuai Xia, Lu Lu, Shibo Jiang, Lize Xiong

**Affiliations:** ^1^ Key Laboratory of Medical Molecular Virology Ministry of Education (MOE)/National Health Commission of China (NHC)/Chinese Academy of Medical Sciences (CAMS), Shanghai Institute of Infectious Disease and Biosecurity, School of Basic Medical Sciences, Fudan University, Shanghai, China; ^2^ Department of Laboratory Medicine, Shanghai Fourth People’s Hospital, School of Medicine, Tongji University, Shanghai, China; ^3^ Shanghai Key Laboratory of Anesthesiology and Brain Functional Modulation, Translational Research Institute of Brain and Brain-Like Intelligence, Shanghai Fourth People’s Hospital, School of Medicine, Tongji University, Shanghai, China; ^4^ Department of Anesthesiology and Perioperative Medicine, Shanghai Fourth People’s Hospital, School of Medicine, Tongji University, Shanghai, China

**Keywords:** SARS-CoV-2 Omicron variant, intensive care, prognostic model, COVID-19, immune biomarkers, cytokines

## Abstract

**Background:**

The severe acute respiratory syndrome coronavirus 2 (SARS-CoV-2) Omicron variant has prevailed globally since November 2021. The extremely high transmissibility and occult manifestations were notable, but the severity and mortality associated with the Omicron variant and subvariants cannot be ignored, especially for immunocompromised populations. However, no prognostic model for specially predicting the severity of the Omicron variant infection is available yet. In this study, we aim to develop and validate a prognostic model based on immune variables to early recognize potentially severe cases of Omicron variant-infected patients.

**Methods:**

This was a single-center prognostic study involving patients with SARS-CoV-2 Omicron variant infection. Eligible patients were randomly divided into the training and validation cohorts. Variables were collected immediately after admission. Candidate variables were selected by three variable-selecting methods and were used to construct Cox regression as the prognostic model. Discrimination, calibration, and net benefit of the model were evaluated in both training and validation cohorts.

**Results:**

Six hundred eighty-nine of the involved 2,645 patients were eligible, consisting of 630 non-ICU cases and 59 ICU cases. Six predictors were finally selected to establish the prognostic model: age, neutrophils, lymphocytes, procalcitonin, IL-2, and IL-10. For discrimination, concordance indexes in the training and validation cohorts were 0.822 (95% CI: 0.748-0.896) and 0.853 (95% CI: 0.769-0.942). For calibration, predicted probabilities and observed proportions displayed high agreements. In the 21-day decision curve analysis, the threshold probability ranges with positive net benefit were 0~1 and nearly 0~0.75 in the training and validation cohorts, correspondingly.

**Conclusions:**

This model had satisfactory high discrimination, calibration, and net benefit. It can be used to early recognize potentially severe cases of Omicron variant-infected patients so that they can be treated timely and rationally to reduce the severity and mortality of Omicron variant infection.

## Introduction

The severe acute respiratory syndrome coronavirus 2 (SARS-CoV-2) Omicron variant has predominantly circulated worldwide, accounting for almost 100% of the newly emerging strains since February 2022 and causing nearly 300 million infections (1). Among all the variants of concern (VOCs), the Omicron variant has exhibited the most mutations (>60 non-synonymous mutations) (2). Most of the mutations are localized in the S protein, especially the receptor-binding domain (RBD), responsible for entering host cells, eliciting immune responses, and acting as the target for drug and neutralizing antibodies (2–4). Such mutations contributed to some unique characteristics of the Omicron variant, such as stronger transmissibility, lower virulence, more remarkable immune evasion, and milder symptoms, including nasal obstruction, fatigue, and sore throat (5). Nonetheless, the severity and fatality brought by the Omicron variant could not be underestimated. During the pandemic wave of the Omicron variant, Hong Kong reported 9,148 deaths as of May 2022, and Japan’s cumulative death toll reached 20,000 till February 2022 (6, 7). Additionally, approximately 2,400 patients with coronavirus disease 2019 (COVID-19) died each day in the United States based on the survey in February 2022 (8). Especially, the elderly, children, neoplasm patients, transplantation recipients, and patients with other comorbidities who had compromised immunity were extremely vulnerable to SARS-CoV-2 infection, and these populations would increase the proportion of severe cases and fatality among infected patients (9). Therefore, how to early recognize underlying severe patients and conduct timely and accurate treatment would favor the prevention of severity progression and reduction of patients’ mortality.

Noteworthy, immunity plays a key role in the occurrence, development, defense, and recovery of SARS-CoV-2 infection. First, innate, humoral, and cellular immunity, e.g., activation of Toll-like receptors, SARS-CoV-2-specific antibodies, plasmacytes, or reactive CD4/CD8^+^ T cells, potently defended against SARS-CoV-2 infection and were tightly associated with disease severity and prognosis (10–12). Second, the Omicron variant infection tended to be mild, and the presence or absence of symptoms was correlated with 1) functional cellular immunity, 2) balance between proinflammatory and anti-inflammatory cytokines, and 3) sera viral-specific IgA, IgM, IgG, or memory B-cell levels (13, 14). Third, immune evasion, one prominent characteristic of the Omicron variant and subvariants, led to decreased efficacy of vaccines and drugs, failed treatment, and the occurrence of severe cases (15, 16). Also, the Omicron variant and subvariants had higher odds of reinfection and breakthrough infection, which are also determined by viral immune evasion and host immune status (17–19). Therefore, immune factors could effectively reflect the severity and prognosis of Omicron variant infection.

For the prediction of COVID-19 patients’ severity and survival, several prognostic models have been established (20–22). However, these models were established based on the results from previously prevailing strains, like Wuhan-Hu-1. The Omicron variant is currently the most dominantly circulating strain worldwide, including subvariants like BA.2.75, XBB, BA.5.2, and BF.7. By its unique etiological features, the Omicron variant differs from the SARS-CoV-2 ancestral strain and other VOCs. Notably, patients with Omicron variant infection have lower hospitalization, intensive care unit (ICU) admission, and mortality rates, as well as shorter rehabilitation time compared with those infected by the SARS-CoV-2 ancestral strain and other VOCs like the Delta variant (23–25). Thus, previously established models may not be applicable to Omicron variant infection. Indeed, no prognostic models anticipated the unique etiological and clinical characteristics of the Omicron variant and were specially developed for Omicron variant-infected COVID-19. Additionally, many countries have terminated COVID-19 quarantine and management, possibly resulting in an expansion of infected populations and a wider pandemic of the Omicron variant in the future. These facts call for the development of a prognostic model that specifically predicts the severity and prognosis of Omicron variant infection.

As suggested above, immune factors are useful indicators of SARS-CoV-2 Omicron variant infection; thus, to predict patients’ severity accurately and sensitively, we aimed to construct a prognostic model based on serial immune factors. To accomplish this, a prognostic study was performed and included COVID-19 patients from the 2022 Omicron subvariant BA.2 epidemic in Shanghai. Clinical and immune indexes were collected, and predictors were selected by different methods to develop the model. The model was assessed from the aspects of discrimination, calibration, and net benefit to ensure its performance.

## Materials and methods

### Participants

This was a single-center prognostic study conducted in Shanghai Fourth People’s Hospital from 12 April 2022 to 17 June 2022. Omicron variant-infected patients were diagnosed and confirmed by the Shanghai Center for Disease Control and Prevention with positive real-time polymerase chain reaction results. Those patients needing further treatment were transferred from temporary treatment centers and admitted to Shanghai Fourth People’s Hospital. Patients were routinely treated according to the Diagnosis and Treatment Scheme of Pneumonia Caused by Novel Coronavirus of China (the ninth version). The eligibility criteria were as follows: 1) having intact basic information to be retrieved (names, gender, ages, and diagnosis) and 2) having examination results of immune cytokines.

### Predictors and outcomes

All the basic clinical information including age, gender, preliminary severity degree when admitted [severity (admitted)], and vaccination was documented upon patients’ hospitalization. Severity (admitted) was judged according to the Diagnosis and Treatment Scheme of Pneumonia Caused by Novel Coronavirus of China (the ninth version): the asymptomatic and light types were defined as the moderate category, and other types were defined as the severe category. After admission, immune-related diagnostic indexes were collected immediately, including 1) absolute numbers of immune cells: leukocytes (×10^9^/L), neutrophils (×10^9^/L), lymphocytes (×10^9^/L), monocytes (×10^9^/L), eosinophils (×10^9^/L), and basophils (×10^9^/L) (BC-75000 Fully Automated Hematology Analyzer, Mindray, Shenzhen, China); 2) inflammatory factors: C-reaction protein (CRP, mg/L) (turbidimetric inhibition immunoassay, Mindray BC-75000 Fully Automated Hematology Analyzer), procalcitonin (PCT, ng/ml) (double-antigen sandwich immunoassay, ECL8000 Automated ECL Immunoassay Analyzer, Lifotronic, Shenzhen, Chnia), serum amyloid A (SAA, mg/L) (turbidimetric inhibition immunoassay, UPPER Automatic Protein Analyzer), and interleukin (IL)-6 (pg/ml) (double-antigen sandwich immunoassay, Lifotronic ECL8000 Automated ECL Immunoassay Analyzer); 3) immune cytokines: IL-17A (pg/ml), IL-10 (pg/ml), interferon (IFN)-γ (pg/ml), IL-2 (pg/ml), IL-1β (pg/ml), IL-5 (pg/ml), IL-12 (pg/ml), IL-8 (pg/ml), IL-4 (pg/ml), and tumor necrosis factor (TNF)-α (pg/ml) (flow cytometry, DxFLEX, Beckman, Bria, USA); and 4) the index reflecting antibody levels: globulin (g/L) (globulin = total protein-albumin, as measured by the Biuret method and the bromocresol green colorimetric method, correspondingly). All the variables listed above were collected before the onset of ICU admission. The outcome of this study was admission to the ICU for the determination of severe cases.

### Statistical analysis

For variables, outliers were identified as values less than 25th percentile minus 1.5-fold of the interquartile range (1.5 × IQR) or more than 75th percentile plus 1.5 × IQR. Outliers were winsorized as 5th percentile or 95th percentile, respectively. Variables with >10% missing values were excluded from the study. Since missing values were missed at random, multiple imputation was applied to variables missing <10% using the R package *mice*, and one imputation result was finally used. Patients were randomly divided into the training cohort (70%) and the validation cohort (30%). Three methods, namely, stepwise regression, least absolute shrinkage and selection operator (LASSO) regression, and best subset selection regression, were used to select predictors in the training cohort. To select variables by stepwise regression, univariate Cox regression was first performed for each variable, and variables with *P*-value <0.1 were included in multivariate Cox regression. Then, the backward stepwise regression was performed, and variables were finally determined by it. In LASSO regression, the variations of partial-likelihood deviance and the coefficients of all the variables with the change of *λ* were studied. When partial-likelihood deviance was lowest, variables with non-zero coefficients included in LASSO regression were selected. In best subset selection regression, variations of *β*, *l*(*β*), Akaike information criterion (AIC), and Bayesian information criterion (BIC) with the changes of the model’s complexity were studied. Predictors in the regression with the minimum AIC were selected.

Eventual predictors were considered based on the results of the three methods and used to establish Cox regression as the optimal model. The model was appraised from three perspectives: discrimination, calibration, and clinical net benefit. Discrimination was determined by the concordance index (C-index) with 95% confidential interval (95% CI) calculated by 1,000 replicates of bootstrap resampling and area under the receiver operating characteristic curve (AUROC). The calibration curves were graphically plotted to evaluate the agreement between predicted probabilities and actual proportions. Net benefit in clinical practice under different threshold probabilities was calculated using the decision curve analysis (DCA) method. A nomogram was displayed for detailed clinical usage of the optimal model. The risk score plot was plotted to display patients’ risk scores in an ascending order, their outcomes with the follow-up time, and the standardized level of each predictor in all patients. For each predictor, the grouping cutoff value was determined by the R package *survminer*, which was used to separate patients into two groups, and the difference between the two groups was evaluated by the Kaplan–Meier method and the log-rank test.

## Results

### Participants

Corresponding to the eligibility criteria, a total of 689 eligible patients were included (**Figure S1**). Among these patients, 59 (8.56%) of them developed into severe cases after hospitalization and were admitted to the ICU (defined as the ICU group), and the remaining 630 (91.44%) patients were not admitted to the ICU until their discharge (defined as the non-ICU group). Patients with intact data accounted for 84.33% with the other 15.67% missing one or more indexes (**Figure S2**). To characterize the patients, baseline characteristics including demographic and clinical features, immune indexes, and outcomes were listed (**Table 1**). Comparisons between the ICU and non-ICU groups revealed several differential variables: age, severity (admitted), leukocytes, neutrophils, lymphocytes, eosinophils, basophils, CRP, SAA, IL-6, PCT, IL-10, IL-2, TNF-α, and globulin. Since the total 689 patients were randomly separated into the training and validation cohorts, comparisons were made between them to investigate whether such separation caused biases in data distribution (**Table S1**). From this table, the *P*-values of all the comparisons were >0.05, indicating that no significant difference existed between the training and validation cohorts.

**Table 1:** Demographic and clinical features, immune indexes, and outcomes of the 689 patients.

Variables	Total (*n* = 689)	Non-ICU (*n* = 630)	ICU (*n* = 59)	*P-*value^a^
Age (years)	76.4 (64.5, 86.7)	75.35 (63.92, 86.3)	86.2 (77.55, 90.35)	<0.001
Gender (*n*)				0.81
Male	308 (45)	283 (45)	25 (42)	
Female	381 (55)	347 (55)	34 (58)	
Severity (admitted) (*n*)				<0.001
Moderate	495 (72)	466 (74)	29 (49)	
Severe	194 (28)	164 (26)	30 (51)	
Leukocytes (×10^9^/L)	5.24 (4.19, 6.81)	5.18 (4.17, 6.66)	7.08 (5.05, 9.73)	<0.001
Neutrophils (×10^9^/L)	3.31 (2.39, 4.58)	3.2 (2.36, 4.33)	5.09 (3.19, 9.31)	<0.001
Lymphocytes (×10^9^/L)	1.21 (0.86, 1.72)	1.27 (0.89, 1.78)	0.9 (0.5, 1.26)	<0.001
Monocytes (×10^9^/L)	0.43 (0.32, 0.57)	0.43 (0.33, 0.56)	0.44 (0.3, 0.58)	0.73
Eosinophils (×10^9^/L)	0.05 (0.01, 0.1)	0.05 (0.02, 0.11)	0.02 (0, 0.05)	<0.001
Basophils (×10^9^/L)	0.01 (0.01, 0.02)	0.02 (0.01, 0.02)	0.01 (0, 0.01)	<0.001
CRP (mg/L)	8.33 (2.93, 23.01)	7.4 (2.67, 20.06)	48.12 (12.2, 110.47)	<0.001
SAA (mg/L)	27.28 (7.87, 107.18)	23.55 (7.31, 85.72)	268.7 (58.32, 320)	<0.001
IL-6 (pg/ml)	28.84 (14.78, 76.66)	27.17 (14.39, 65.79)	48.88 (27.81, 154.95)	<0.001
PCT (ng/ml)	0.02 (0.02, 0.06)	0.02 (0.02, 0.05)	0.45 (0.05, 0.45)	<0.001
IL-17A (pg/ml)	1.07 (0.33, 2.66)	1.02 (0.32, 2.58)	1.5 (0.49, 3)	0.29
IL-10 (pg/ml)	4.28 (2.38, 7.56)	4.12 (2.33, 6.9)	8.12 (3.83, 12.48)	<0.001
IFN-γ (pg/ml)	1.73 (0.4, 5.68)	1.82 (0.41, 5.5)	1.27 (0.33, 5.89)	0.55
IL-2 (pg/ml)	0.08 (0.04, 0.88)	0.08 (0.04, 0.88)	0.1 (0.06, 15.12)	0.01
IL-1β (pg/ml)	0.89 (0.27, 2.03)	0.89 (0.27, 2.02)	0.89 (0.27, 2.5)	0.72
IL-5 (pg/ml)	0.07 (0.04, 0.14)	0.07 (0.04, 0.12)	0.08 (0.04, 1.64)	0.36
IL-12 (pg/ml)	0.07 (0.04, 0.5)	0.07 (0.04, 0.49)	0.07 (0.04, 0.62)	0.66
IL-8 (pg/ml)	101.7 (30.49, 239.7)	104.11 (30.24, 244.74)	94.09 (39.42, 180.94)	0.36
IL-4 (pg/ml)	0.7 (0.07, 2.91)	0.7 (0.07, 3.03)	0.68 (0.08, 1.65)	0.78
TNF-α (pg/ml)	5.98 (2.62, 12.25)	6.35 (2.77, 12.87)	3.25 (1.02, 7.14)	<0.001
Globulin (g/L)	22.25 (19.83, 25.11)	22.05 (19.65, 24.85)	23.89 (21.52, 26.52)	0.002

a For normally distributed continuous variables, the unpaired t-test (two-tailed) was used; for non-normally distributed continuous variables, the Wilcoxon signed-rank test (two-tailed) was used; for categorical variables, Pearson’s chi-squared test (two-tailed) was used.

### Predictor selections and verification

**Figure 1:**
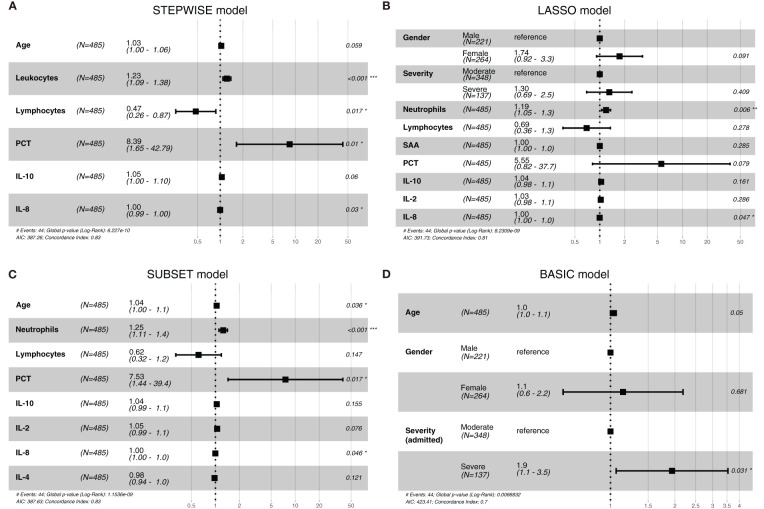
The forest plots of the STEPWISE, least absolute shrinkage and selection operator (LASSO), SUBSET, and BASIC models. Cox regression results of the STEPWISE model **(A)**, LASSO model **(B)**, SUBSET model **(C)**, and BASIC model **(D)** were displayed in the forest plots as HR values with 95% CI and *P*-values.

**Figure 2:**
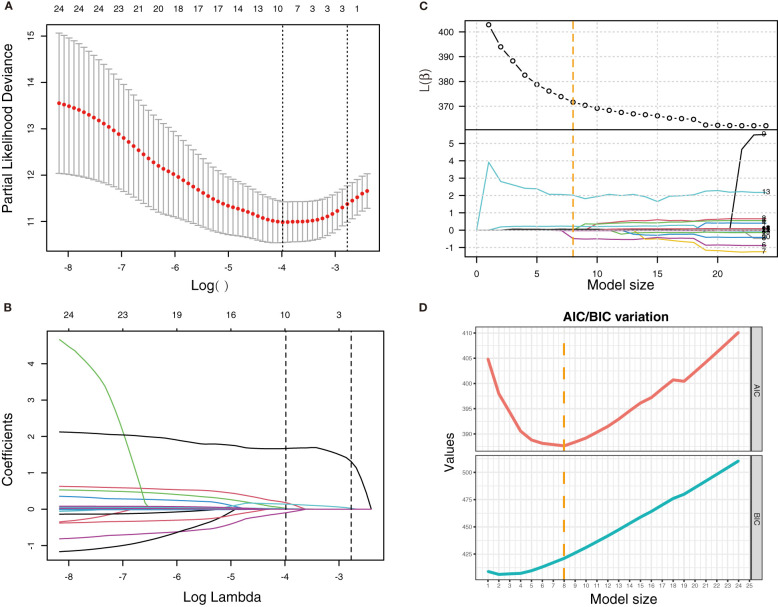
LASSO regression and best subset selection regression for the selection of variables. **(A)** Variation of partial-likelihood deviance with the change of log(*λ*) in LASSO regression. **(B)** Coefficient of each variable in LASSO regression with the change of log(*λ*). First vertical dotted line: *λ* value (*λ*_min_) when partial-likelihood deviance was minimum. Second vertical dotted line: *λ*_min_ + standard error (*λ*_1se_). **(C)** Variation of *β* and *l*(*β*) with the change of model size (numbers of variables included in the model). **(D)** Variation of AIC and BIC with the change of model size. Vertical dotted line: model size when AIC was minimum.

*Via* stepwise regression (**Table 2**), six variables were eventually retained in the regression, namely, age, leukocytes, lymphocytes, PCT, IL-10, and IL-8, and they were used to construct Cox regression named the STEPWISE model (**Figure 1A**). In LASSO regression (**Figures 2A, B**), when partial-likelihood deviance was lowest, variables with non-zero coefficient were gender, severity (admitted), neutrophils, lymphocytes, SAA, PCT, IL-10, IL-2, and IL-8. They were included in Cox regression named the LASSO model (**Figure 1B**). In best subset selection regression (**Figures 2C, D**), age, neutrophils, lymphocytes, PCT, IL-10, IL-2, IL-8, and IL-4 were included when the AIC was minimum. Cox regression established using these variables was named the SUBSET model (**Figure 1C**). Moreover, to verify whether introducing immune predictors into the prognostic model would improve the predictive efficacy, a model named the BASIC model, only including basic demographic and clinical variables (age, gender, and severity (admitted)), was established and compared with the STEPWISE, LASSO, and SUBSET models (**Figure 1D**).

**Table 2:** Results of univariate and multivariate Cox regression calculating HR values for ICU admission before stepwise regression.

	Univariate		Multivariate	
Variables	*P-*value	HR (95% CI)	*P-*value	HR (95% CI)
Age (years)	0.01	1.04 (1.01~1.07)	0.15	1.03 (0.99~1.06)
Gender: female	0.16	1.56 (0.85~2.86)		
Severity (admitted): severe	0.008	2.22 (1.23~4.01)	0.23	1.48 (0.78~2.81)
Leukocytes (×10^9^/L)	<0.001	1.27 (1.13~1.42)	0.46	1.2 (0.74~1.96)
Neutrophils (×10^9^/L)	<0.001	1.35 (1.21~1.5)	0.89	1.04 (0.63~1.71)
Lymphocytes (×10^9^/L)	0.003	0.38 (0.2~0.72)	0.12	0.5 (0.2~1.2)
Monocytes (×10^9^/L)	0.91	1.09 (0.24~4.94)		
Eosinophils (×10^9^/L)	0.11	0.01 (0~2.85)		
Basophils (×10^9^/L)	0.04	0 (0~0.2)	0.73	471.58 (0~4.1 × 10^17^)
CRP (mg/L)	<0.001	1.01 (1.01~1.02)	0.31	0.99 (0.98~1.01)
SAA (mg/L)	<0.001	1.01 (1~1.01)	0.36	1 (1~1.01)
IL-6 (pg/ml)	0.34	1 (1~1)		
PCT (ng/ml)	<0.001	50.43 (11.84~214.82)	0.03	9.64 (1.21~77.05)
IL-17A (pg/ml)	0.59	1.02 (0.95~1.1)		
IL-10 (pg/ml)	0.002	1.08 (1.03~1.13)	0.29	1.03 (0.97~1.09)
IFN-γ (pg/ml)	0.41	1.01 (0.98~1.05)		
IL-2 (pg/ml)	0.04	1.05 (1~1.1)	0.17	1.04 (0.98~1.09)
IL-1β (pg/ml)	0.25	1.06 (0.96~1.17)		
IL-5 (pg/ml)	0.66	1.1 (0.73~1.65)		
IL-12 (pg/ml)	0.57	0.89 (0.6~1.33)		
IL-8 (pg/ml)	0.03	1 (1~1)	0.05	1 (0.99~1)
IL-4 (pg/ml)	0.76	1 (0.97~1.02)		
TNF-α (pg/ml)	0.17	0.98 (0.94~1.01)		
Globulin (g/L)	0.06	1.08 (1~1.16)	0.44	1.03 (0.95~1.13)

**Figure 3:**
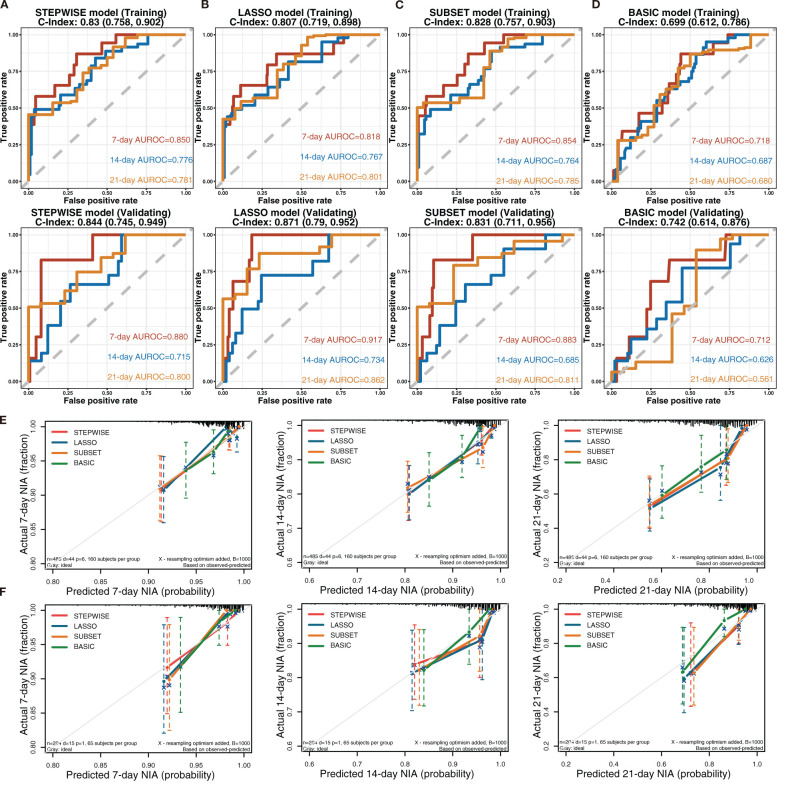
Discrimination and calibration of the STEPWISE, LASSO, SUBSET, and BASIC models. **(A–D)** Receiver operating characteristic (ROC) curves of the STEPWISE model **(A)**, LASSO model **(B)**, SUBSET model **(C)**, and BASIC model **(D)** with AUROC and C-indexes with 95% CI. Red: the 7-day ROC curve; blue: the 14-day ROC curve, yellow: the 21-day ROC curve. **(E, F)** Calibration plots of 7, 14, and 21 days displaying the relationship between predicted no ICU admission (NIA) probabilities and actual NIA proportions in the training **(E)** and validation **(F)** cohorts. Red: the STEPWISE model; blue: the LASSO model; yellow: the SUBSET model; green: the BASIC model.

**Figure 4:**
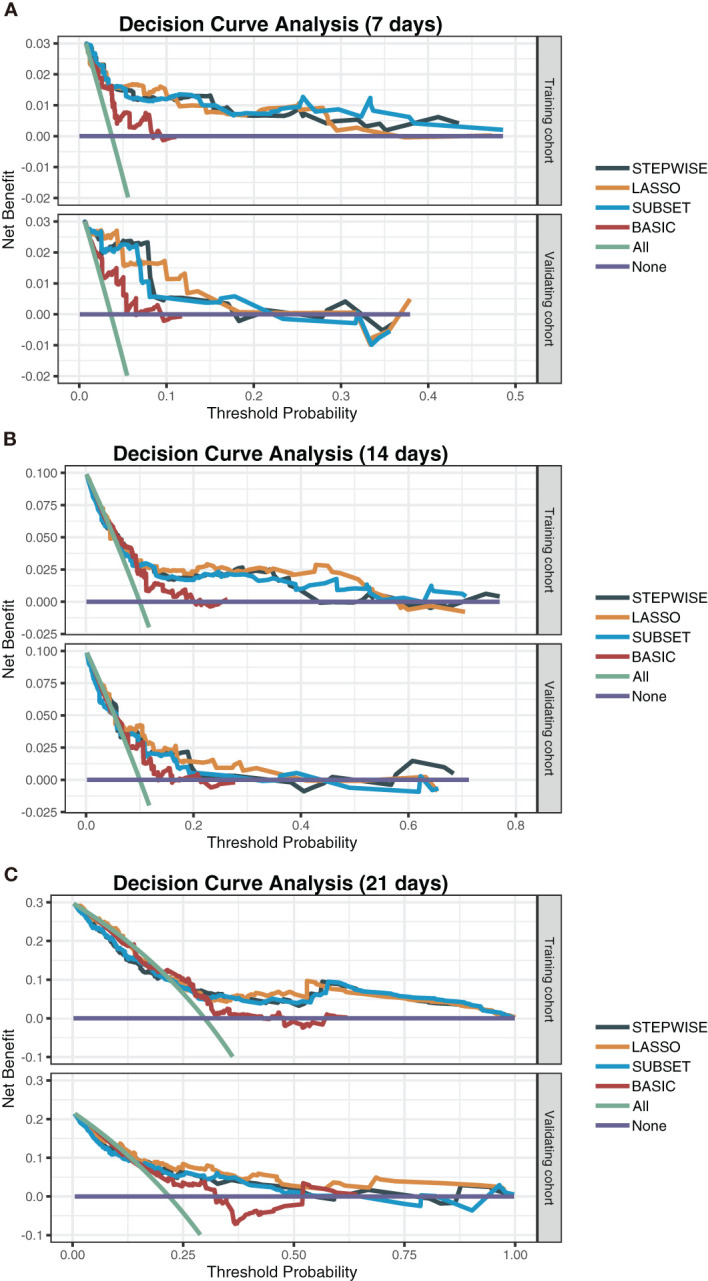
Decision curve analysis of the STEPWISE, LASSO, SUBSET, and BASIC models. Decision curves of the four models showing the net benefit of using each model according to different threshold probabilities in both training and validation cohorts. **(A)** The 7-day decision curves. **(B)** The 14-day decision curves. **(C)** The 21-day decision curves. Gray: the STEPWISE model; yellow: the LASSO model; blue: the SUBSET model; brown: the BASIC model; green: all patients receiving treatment; purple: no patient receiving treatment.

To observe the reliability of predictors selected by these methods, the performance of the three models was firstly evaluated. First, the models’ C-indexes with 95% CI and AUROC were calculated (**Figures 3A–D**). Accordingly, the C-indexes of the STEPWISE, LASSO, and SUBSET models and the majority of their 7-, 14-, and 21-day AUROCs were over 0.8, and all the values were higher than those of the BASIC model. Thus, all three models had quite satisfactory discrimination which was apparently better than that of the BASIC model. Then, the models’ calibration in the training and validation cohorts was displayed (**Figures 3E, F**). All the models’ calibration curves closely approached the diagonal edge (ideal line), indicating that their predicted probabilities of no ICU admission (NIA) were in agreement with actual NIA proportions. In the DCA curves of 7, 14, and 21 days, the STEP, LASSO, and SUBSET models had more net benefit in a wider range compared with the BASIC model (**Figure 4**). Conclusively, the STEP, LASSO, and SUBSET models exhibited good performance in discrimination, calibration, and net benefit, and therefore, predictors selected by their corresponding methods were reliable in predicting ICU admission. Meanwhile, all three models had obviously better performance than the BASIC model, demonstrating that introducing immune variables into the model could improve the model’s predictive ability.

### Model development and validation

**Figure 5:**
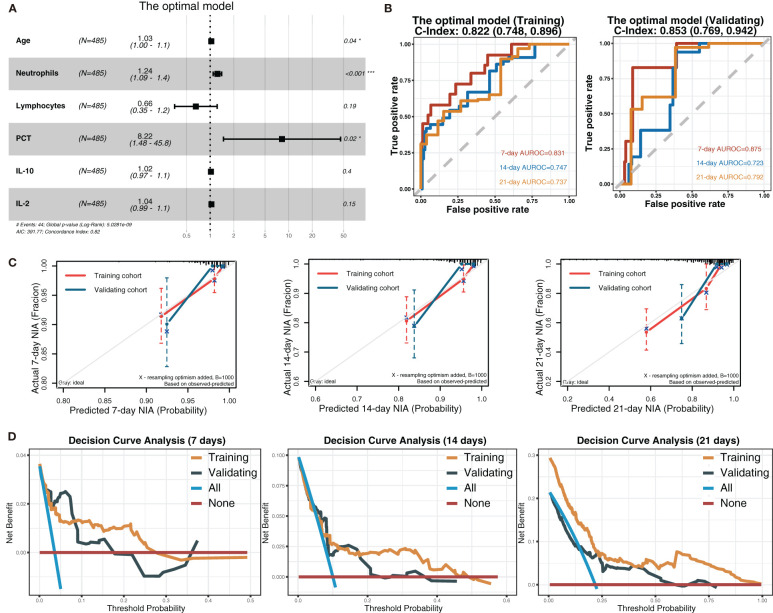
The optimal model and its performance evaluation. **(A)** The forest plot displaying Cox regression results of the optimal model as HR values with 95% CI and *P*-values. **(B)** ROC curves of the optimal model with AUROC and C-indexes with 95% CI. Red: the 7-day ROC curve; blue: the 14-day ROC curve, yellow: the 21-day ROC curve. **(C)** Calibration plots of 7, 14, and 21 days displaying the relationship between predicted NIA probabilities and actual NIA proportions. Red: the training cohort; blue: the validation cohort. **(D)** Decision curves of the optimal model showing the net benefit under different threshold probabilities in 7, 14, and 21 days. Yellow: the training cohort; black: the validation cohort; blue: all patients receiving treatment; brown: no patient receiving treatment.

Considering all predictors selected by the three models, we chose predictors emerging more than twice to be included in the eventually optimal model: age, neutrophils, lymphocytes, PCT, IL-10, IL-2, and IL-8. Additionally, IL-8 was removed because of its insignificant HR value (1 (1, 1)). Cox regression was developed using the remaining six variables as the optimal model (**Figure 5A**). Afterward, its performance was assessed. The optimal model’s C-indexes in the training and validation cohorts were more than 0.8, and its AUROCs of 7, 14, and 21 days were over 0.7, suggesting its excellent discrimination (**Figure 5B**). In its calibration plot, the predicted NIA probabilities were proximal to actual NIA proportions (**Figure 5C**). From 7 to 14 days and then to 21 days in the DCA curves (**Figure 5D**), the ranges of threshold probability with positive net benefit extended over time, and in the 21-day DCA curves, the ranges in the training and validation cohorts were 0~1 and nearly 0~0.75, retrospectively, suggesting a considerable clinical benefit of the model.

### Model specification

**Figure 6:**
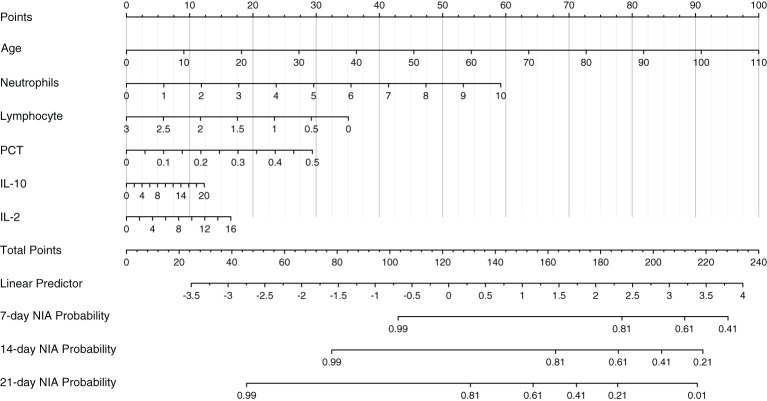
The nomogram of the optimal model. Values in the scale ruler of each variable corresponded to their points in the first line. A summary of these points was displayed as the total points, and the total points corresponded to a patient’s NIA probability in 7, 14, and 21 days.

**Figure 7:**
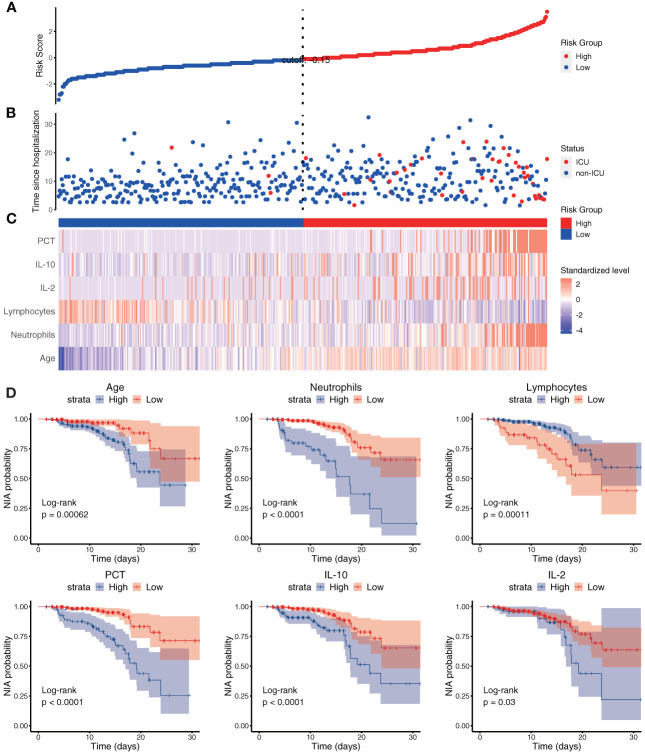
The risk score and each variable’s risk contribution in the optimal model. **(A–C)** The *X*-axes represented all the patients ranked by their risk scores calculated from the optimal model. **(A)** The *Y*-axis displayed the detailed risk score of each patient; **(B)** the *Y*-axis displayed the follow-up time since admission along with the patients’ outcomes; **(C)** heatmap showing the standardized level of each variable in all patients. **(D)** Kaplan–Meier survival curves of variables in the optimal model with *P*-values of the log-rank test. Blue: the high-value group; red: the low-value group.

Based on Cox regression of the optimal model, a nomogram was developed to calculate the detailed NIA probability in 1, 2, and 3 weeks after hospitalization (**Figure 6**). According to the risk score plot, ICU-admitted patients appeared intensively in the high-risk groups with correspondingly higher risk scores (**Figures 7A, B**). Moreover, higher values of age, neutrophils, PCT, IL-2, and IL-10 appeared in patients with higher risk scores, and higher values of lymphocytes appeared in patients with lower risk scores, indicating their specific promotive or inhibitive functions in ICU admission for patients (**Figure 7C**). The significant differences between groups with high and low values were also proven by the Kaplan–Meier method and the log-rank test, and all the predictors’ *P*-values in the log-rank test were less than 0.05 (**Figure 7D**).

## Discussion

Since December 2021, the SARS-CoV-2 Omicron variant has rapidly preempted the shares of other VOCs like the Delta and Gamma variants and become the predominant variant globally. The enhanced ability of the Omicron variant to evade vaccine or infection-induced immunity and bind with angiotensin-converting enzyme 2 receptor confers its exceedingly potent infectivity and high odds of reinfection and breakthrough infection (18, 26, 27). Moreover, more and more countries canceled their previously strict COVID-19 management, thus leading to a surge of infected cases. Considering that newly infected patients mainly have symptoms like fever, fatigue, runny nose, and sore throat, the key point of handling COVID-19 becomes how to recognize potentially severe cases and prevent illness progression, especially in immunocompromised and vulnerable populations. To address this problem, we aimed to construct a prognostic model to early predict an Omicron variant-infected patient’s probability of developing into a severe case using immune-related predictors. First, three methods were used to select the appropriate predictors to establish a model. Then, the model was assessed from aspects of discrimination, calibration, and net benefit. Also, a nomogram was given for detailed use.

The optimal model established by us provided several meaningful implications. First, host immunity plays a critical role in the occurrence, development, and defense of SARS-CoV-2 infection. Early and coordinated immune responses are tightly associated with effective viral clearance and milder symptoms in COVID-19 patients; however, dysregulated and delayed immune responses induce serious pulmonary damage, sepsis, cytokine storm, and even multiorgan failure (28–30). Therefore, immune factors could directly and powerfully reflect the severity and prognosis of COVID-19, and they were used in our study to maximize the model’s predictive power. Many of the previous studies have focused on other kinds of predictors like saturation of oxygen, estimated glomerular filtration rate, dyspnea, d-dimer, prothrombin time, NT-proBNP, and myoglobin, while no studies constructed prognostic models specially from the aspect of immunity (21, 22, 31). Second, the number of Omicron variant-infected patients would increase continuously with the evolution and transmission of the Omicron variant over time. Given the huge proportion (over one-third) of the elderly and children in the world, along with other immunocompromised populations, this prognostic model is expected to have promising prospects for clinical application. Third, values of all the predictors in this study were collected immediately after the patients’ admission; therefore, it will facilitate the early recognition of the potential severe cases upon their hospitalization and allow the timely initiation of suitable treatment. It would also benefit the rational allocation of medical resources to maximize their usage. The examination of these six predictors is inexpensive and easy to apply. Additionally, this prognostic model is particularly developed for the Omicron variant and subvariants, thus being more applicable than previous models based on the ancestral strain or other VOCs.

In our prognostic model, higher levels of neutrophils, PCT, IL-2, and IL-10 and lower levels of lymphocytes, along with advanced age, were considered factors contributing to ICU admission. Neutrophils, the main driver of innate immunity, could eliminate SARS-CoV-2 *via* phagocytosis, extracellular traps, and cytokine release, and it could also result in hyperinflammation and immunopathological damage in COVID-19 patients, which was tightly associated with patients’ severity and survival (32). PCT was an immune factor elevated after bacterial, fungal, and parasitic infection, reflective of serious infection and sepsis (33). Its apparently promotive effect on ICU admission of Omicron variant-infected patients [HR with 95% CI: 11.72 (2.29~60.06)] suggests that comorbid infection or inflammation is an important factor exacerbating the prognosis of Omicron variant infection. Actually, comorbid bacterial infection was common in COVID-19 patients due to the damaged functions of T cells, B cells, and NK cells caused by SARS-CoV-2 infection, intubation treatment, or basically compromised immunity, which could aggravate systemic inflammation and increase disease severity and death rate, as previously reported (34–36). IL-2, a stimulator of T-cell proliferation and effector/memory T-cell production, is a proinflammatory cytokine. Its expression level was elevated after SARS-CoV-2 infection and associated with disease severity (37, 38). IL-10, a multifunctional cytokine modulating many cytokine releases and immune cell functions, is also a promotive factor for critical illness of COVID-19 (39, 40). It was reported that the levels of risk factors like IL-2, IL-6, IL-7, IL-10, and TNF-α in the patients infected with the Wuhan-Hu-1, Alpha variant, Delta variant, or Omicron variant were different (41). Here, we also noticed that the levels of IL-2 and IL-10 in severe Omicron variant-infected patients were lower than those in Wuhan-Hu-1-infected patients (42). These findings suggest that these factors’ contribution to the severity of patients infected with different SARS-CoV-2 variants may be different because of their different virulence and pathogenicity. Therefore, it is essential to develop different prognostic models based on immune variables in patients infected with the SARS-CoV-2 ancestral strain or other VOCs.

Certainly, some limitations existed in our study. First, considering the 10 events per variable criterion of sample sizes, a total of 60 ICU-admitted patients were needed in this Cox regression. The training cohort had 44 ICU-admitted patients, lower than the satisfactory sample size. However, since ICU admission is a low-frequency event in the Omicron epidemic area, such sample size is still acceptable. Second, many patients lacked examination results of immune cytokines, and thus, a selection bias might exist. Third, patients in our study were mainly infected by Omicron subvariant BA.2, thus being unable to comprehensively include more patients infected by other Omicron subvariants (43). However, the Omicron subvariants’ pathogenic, antigenic, and immune properties have many similarities, and therefore, the differences in immune responses induced by them are limited (44, 45). To solve these issues, a further external validation study containing sufficient samples, less missing values, and patients infected by different Omicron subvariants is warranted.

In general, we have developed and validated a prognostic model to predict the severity of Omicron variant infection based on six predictors: older age, higher numbers of neutrophils, lower numbers of lymphocytes, and higher levels of PCT, IL-2, and IL-10. This prognostic model has high discrimination, calibration, and net benefit with good potential for a wide clinical application.

## Data availability statement

The raw data supporting the conclusions of this article will be made available by the authors, without undue reservation.

## Ethics statement

The study involving human participants was reviewed and approved by the Ethics Committee of Shanghai Fourth People’s Hospital (approval number: 2022098-001). Written informed consent to participate in this study was provided by the participants’ legal guardian/next of kin.

## Author contributions

LX, SJ, and LL had access to all the data in the study and take responsibility for the integrity of the data and the accuracy of data analysis. Concept and design: LX, SJ, LL, and TL. Acquisition, analysis, or interpretation of data: TL, QM, XY, and SX. Drafting of the manuscript: TL. Critical revision of the manuscript for important intellectual content: All authors. Statistical analysis: TL. Obtained funding: SX, SJ, and LL. Administrative, technical, or material support: LX, SJ, and LL. Supervision: LX, SJ, and LL. All authors contributed to the article and approved the submitted version.
