# Supplementary figures and images for: Development and validation of a prognostic model based on immune variables to early predict severe cases of SARS-CoV-2 Omicron variant infection
Source: Front Immunol. 2023 Mar 1;14:1157892. doi: 10.3389/fimmu.2023.1157892 (PMC10014461; doi:10.3389/fimmu.2023.1157892)

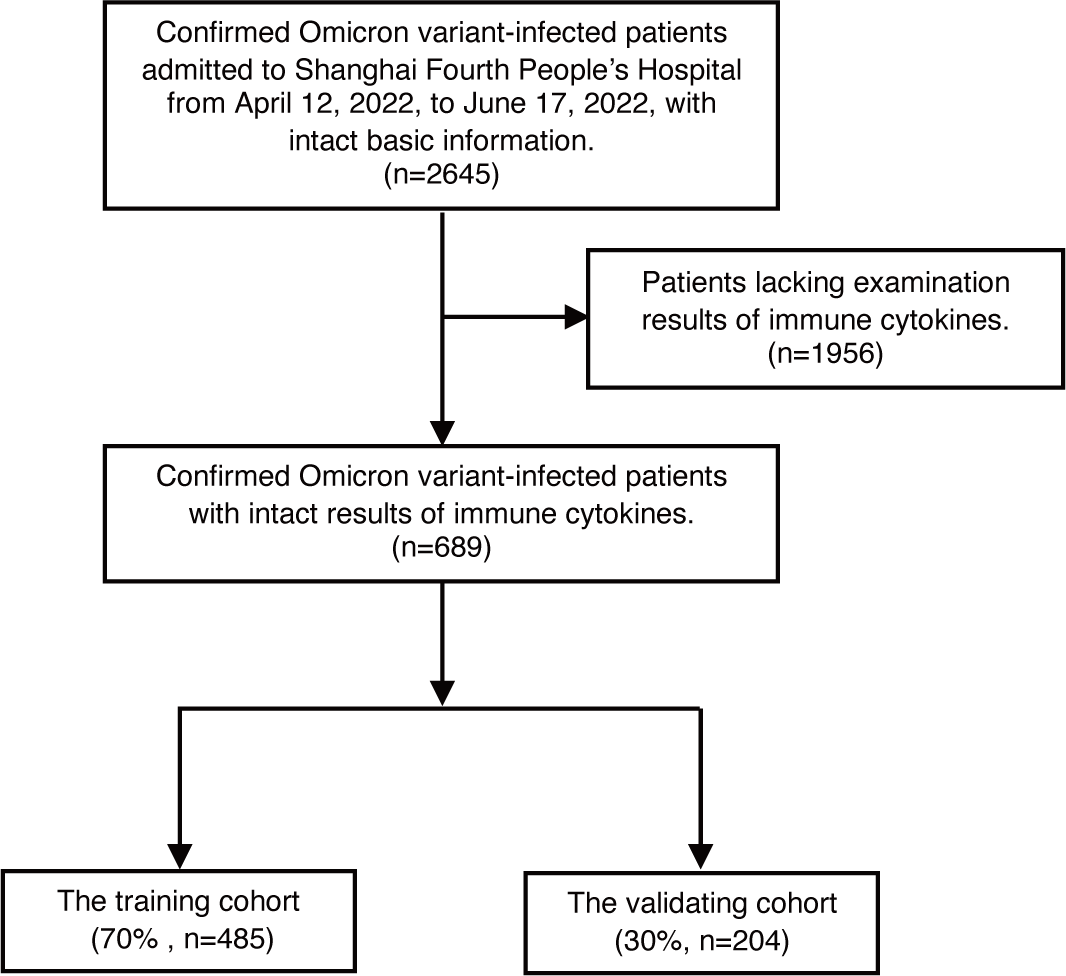

Supplement: Supplementary file 1 [file Image_1.tif]

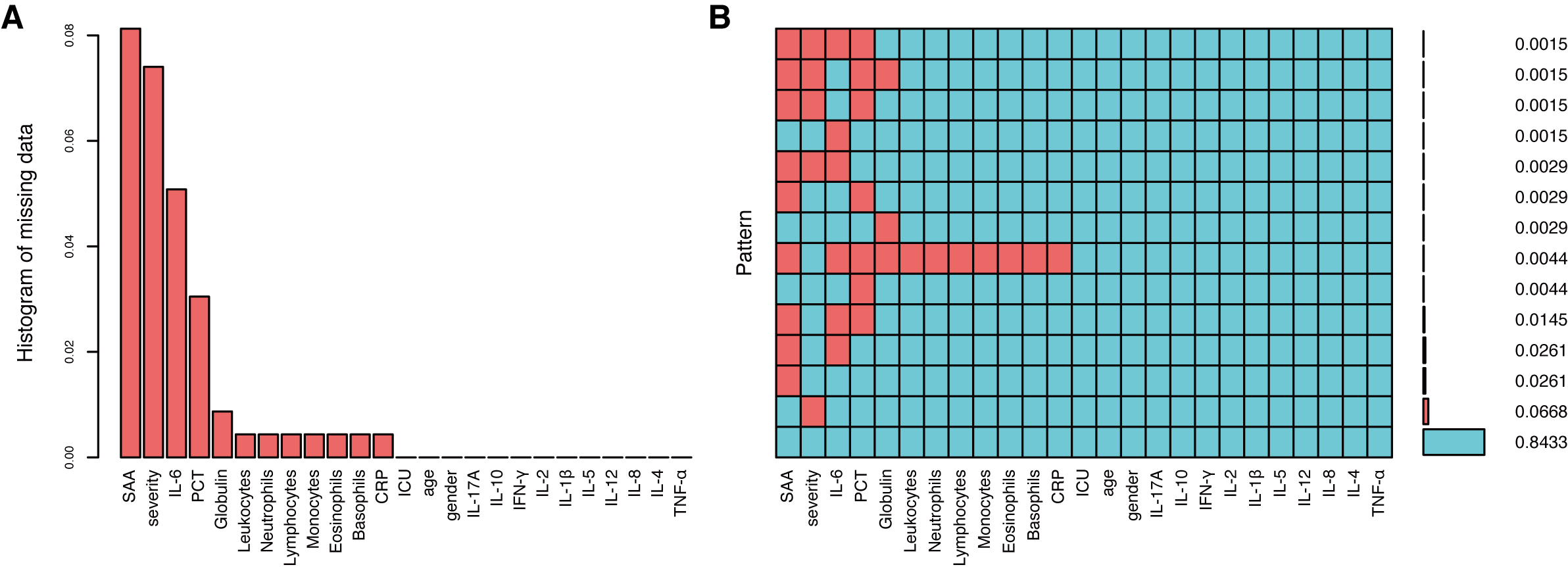

Supplement: Supplementary file 2 [file Image_2.tif]
