# Supplementary material for: Development and validation of a prognostic model based on immune variables to early predict severe cases of SARS-CoV-2 Omicron variant infection
Source: Front Immunol. 2023 Mar 1;14:1157892. doi: 10.3389/fimmu.2023.1157892 (PMC10014461; doi:10.3389/fimmu.2023.1157892)
Supplement: Supplementary file 3 [file DataSheet_1.docx]

**Supplementary materials**

**Supplementary tables**

**Table S1.** Comparisons between training and validating cohorts.

**Supplementary figures**

**Figure S1. The detailed including and excluding criteria for participant selection.** The including criteria were 1) confirmed Omicron variant-infected patients; 2) patients being admitted to Shanghai Forth People’s Hospital from April 12, 2022, to June 17, 2022; 3) with intact basic information (names, gender, ages, and diagnosis). The excluding criterion was lacking examination results of immune cytokines. The finally eligible patients were randomly separated into the training (70%) and validating cohorts (30%).

**Figure S2. Detailed situation of data missing in this study.** The variables missing more than 10% were removed from this study, and variables with less than 10% missing were displayed in this figure. (A) The proportion of each variable’s missing values in the whole data. (B) All the missing situations with the percentage of patients in each situation. Blue: intact variables; red: missing variables.
