# Supplementary material for: Development and validation of a prognostic model based on immune variables to early predict severe cases of SARS-CoV-2 Omicron variant infection
Source: Front Immunol. 2023 Mar 1;14:1157892. doi: 10.3389/fimmu.2023.1157892 (PMC10014461; doi:10.3389/fimmu.2023.1157892)
Supplement: Supplementary file 5 [file Table_1.docx]

**Table S1. Comparisons between training and validating cohorts.**

| **Variables** | **Total (n = 689)** | **The Training cohort (n = 485)** | **The Validating cohort (n = 204)** | ***P-*value^a^** |
| --- | --- | --- | --- | --- |
| ICU (n) |  |  |  | 0.56 |
| Non-ICU group | 630 (91) | 441 (91) | 189 (93) |  |
| ICU group | 59 (9) | 44 (9) | 15 (7) |  |
| Age (year) | 76.4 (64.5, 86.7) | 76.3 (64.4, 87.1) | 76.8 (64.97, 86.3) | 0.72 |
| Gender (n) |  |  |  | 0.54 |
| Male | 308 (45) | 221 (46) | 87 (43) |  |
| Female | 381 (55) | 264 (54) | 117 (57) |  |
| Severity (admitted) (n) |  |  |  | 0.69 |
| Moderate | 492 (71) | 349 (72) | 143 (70) |  |
| Severe | 197 (29) | 136 (28) | 61 (30) |  |
| Leukocytes (×10^9^/L) | 5.24 (4.2, 6.82) | 5.19 (4.26, 6.81) | 5.33 (4.03, 6.87) | 0.86 |
| Neutrophils (×10^9^/L) | 3.31 (2.4, 4.6) | 3.31 (2.41, 4.52) | 3.34 (2.35, 4.69) | 0.98 |
| Lymphocytes (×10^9^/L) | 1.21 (0.86, 1.73) | 1.21 (0.84, 1.72) | 1.22 (0.92, 1.74) | 0.60 |
| Monocytes (×10^9^/L) | 0.43 (0.32, 0.57) | 0.42 (0.33, 0.57) | 0.45 (0.32, 0.58) | 0.84 |
| Eosinophils (×10^9^/L) | 0.04 (0.01, 0.1) | 0.05 (0.01, 0.1) | 0.04 (0.01, 0.1) | 0.77 |
| Basophils (×10^9^/L) | 0.01 (0.01, 0.02) | 0.01 (0.01, 0.02) | 0.01 (0.01, 0.02) | 0.45 |
| CRP (mg/L) | 8.33 (2.93, 23.64) | 7.82 (2.85, 22.77) | 9.26 (3.03, 26.25) | 0.54 |
| SAA (mg/L) | 28.17 (7.81, 104.71) | 29.54 (8.33, 107.18) | 25.62 (7.12, 96.02) | 0.36 |
| IL-6 (pg/ml) | 28.84 (14.58, 79.98) | 28.61 (14.68, 74.1) | 30.74 (14.29, 93.71) | 0.85 |
| PCT (ng/ml) | 0.02 (0.02, 0.06) | 0.02 (0.02, 0.06) | 0.02 (0.02, 0.06) | 0.36 |
| IL-17A (ng/ml) | 1.07 (0.33, 2.66) | 1.09 (0.39, 2.56) | 1.02 (0.23, 2.88) | 0.35 |
| IL-10 (ng/ml) | 4.28 (2.38, 7.56) | 4.33 (2.57, 7.76) | 4.12 (2.13, 6.78) | 0.11 |
| IFN-γ (ng/ml) | 1.73 (0.4, 5.68) | 1.73 (0.43, 5.55) | 1.75 (0.38, 5.77) | 0.80 |
| IL-2 (ng/ml) | 0.08 (0.04, 0.88) | 0.08 (0.04, 0.99) | 0.07 (0.04, 0.77) | 0.43 |
| IL-1β (ng/ml) | 0.89 (0.27, 2.03) | 0.94 (0.34, 2.01) | 0.84 (0.19, 2.26) | 0.20 |
| IL-5 (ng/ml) | 0.07 (0.04, 0.14) | 0.07 (0.04, 0.18) | 0.07 (0.04, 0.1) | 0.79 |
| IL-12 (ng/ml) | 0.07 (0.04, 0.5) | 0.07 (0.04, 0.5) | 0.07 (0.04, 0.44) | 0.91 |
| IL-8 (ng/ml) | 101.7 (30.49, 239.7) | 107.08 (35.99, 251.52) | 86.65 (19.93, 217.21) | 0.07 |
| IL-4 (ng/ml) | 0.7 (0.07, 2.91) | 0.77 (0.07, 2.82) | 0.56 (0.07, 3.07) | 0.61 |
| TNF-α (ng/ml) | 5.98 (2.62, 12.25) | 5.95 (2.63, 12.29) | 6.05 (2.52, 11.33) | 0.51 |
| Globulin (g/L) | 22.24 (19.83, 25.13) | 22.12 (19.61, 24.85) | 22.74 (20.37, 25.73) | 0.09 |

^a^ For normally distributed continuous variables, unpaired t test (two-tailed) was used; for nonnormally distributed continuous variables, Wilcoxon signed-rank test (two-tailed) was used; for categorical variables, Pearson’s chi-squared test (two-tailed) was used.
